# Group Assessments to Help Build Online Learning Communities in Biomedical Science Distance Learning Programmes

**DOI:** 10.3389/bjbs.2023.11891

**Published:** 2023-12-15

**Authors:** Beverley C. Millar, Harriet Purkis, John E. Moore, Stephen McClean, Colm J. Lowery

**Affiliations:** ^1^ School of Biomedical Sciences, Ulster University, Coleraine, United Kingdom; ^2^ Northern Ireland Public Health Laboratory, Belfast City Hospital, Belfast, United Kingdom; ^3^ Department of Hospitality and Tourism Management, Ulster University, Coleraine, United Kingdom

**Keywords:** debate, distance learning, digital skills, e-learning, group work, learning communities, online, microbiology

## Abstract

**Introduction:** Biomedical Science distance learning courses offer flexibility in study while in employment. Asynchronous and self-learning approaches are common within such courses and often student-student interaction is limited. The aims of this study were to establish learning communities, develop confidence in participating in online teamwork and foster an appreciation of transferable skills including digital capabilities through remote group activities.

**Materials and Methods:** Two cohorts of students (*n* = 20/*n* = 21) were enrolled in a microbiology module of an IBMS accredited MSc distance learning course. Groups of 4–5 students produced a digital output relating to current global infection-related issues, namely, assignment 1, production of a slide deck, which peers could use as learning resources and assignment 2, a voiceover PowerPoint debate, and infographic, voting assessment and peer/self-marking. Students also prepared reflections using written format and a FlipGrid video-recording. A qualitative content analysis was conducted on reflections from all students. Students completed a pre- and post-assignment survey focused on the development of transferable skills for the biomedical sector.

**Results:** Students’ skills and confidence increased following completion of the group assignment, as evident from the pre- and post-questionnaire responses, namely, possession of digital skills and digital creation abilities (29% v 83%), applying for jobs which require digital skills (54% v 89%), talking about examples of using digital media during job interviews (21% v 78%) and demonstration of creativity during assignment tasks (33% v 90%). Critical thinking was more commonly demonstrated during the debate in comparison to the slide deck activity (*p* = 0.001). The importance of developing digital skills, was higher following completion of the group activities (*p* = 0.03). Students reflected on the value of the group activities in relation to knowledge acquisition (85%, 86%), collegiality (70%, 71%), digital skills development (80%, 90%), the fact that the activities were enjoyable (70%, 67%) and the development of peer interaction and support (50%, 67%) in relation to assignment 1 and 2, respectively.

**Discussion:** Increasingly digital technologies are being used in the healthcare sector resulting in updated HCPC Standards of Proficiency. This study highlights that virtual group activities promote the establishment of supportive learning communities and the development of transferable skills including digital capabilities for application within the biomedical science workplace.

## Introduction

The origins of education delivered by distance learning trace back to the 18th century, when on 20 March 1728, the *Boston Gazette*, advertised teaching and tutoring on the subject of shorthand, which Professor Caleb Phillips delivered by correspondence [1]. Numerous authors have described the history of distance learning in depth [1, 2] but it is of interest to note that, within the United Kingdom, during the 1840s, Sir Isaac Pitman established the first correspondence teaching school. Pitman delivered not only shorthand teaching, but also assessment and feedback using distance learning as he sent postcards to students on to which they would transcribe passages from the Bible and return, by post, for correction [2, 3]. Globally, correspondence education expanded, during the 19th century, with a significant event occurring in 1858, when the University of London pioneered the delivery of global degree level education by correspondence distance learning education [4]. The most subsequent notable development over 100 years later in relation distance learning occurred on the 23 April 1969, when the Open University (OU) was established by the Royal Charter [4]. According to Royal Charter “*The objects of the University shall be the advancement and dissemination of learning and knowledge by teaching and research by a diversity of means such as broadcasting and technological devices appropriate to higher education, by correspondence tuition, residential courses and seminars and in other relevant ways, and shall be to provide education of University and professional standards for its students and to promote the educational wellbeing of the community generally*” [5]. The OU became the world’s first university solely devoted to distance learning and in its first year, 1971, enrolled approximately 24,000 students, offering courses to students with all abilities and disabilities which were primarily delivered by correspondence and television [1].

**FIGURE 1:**
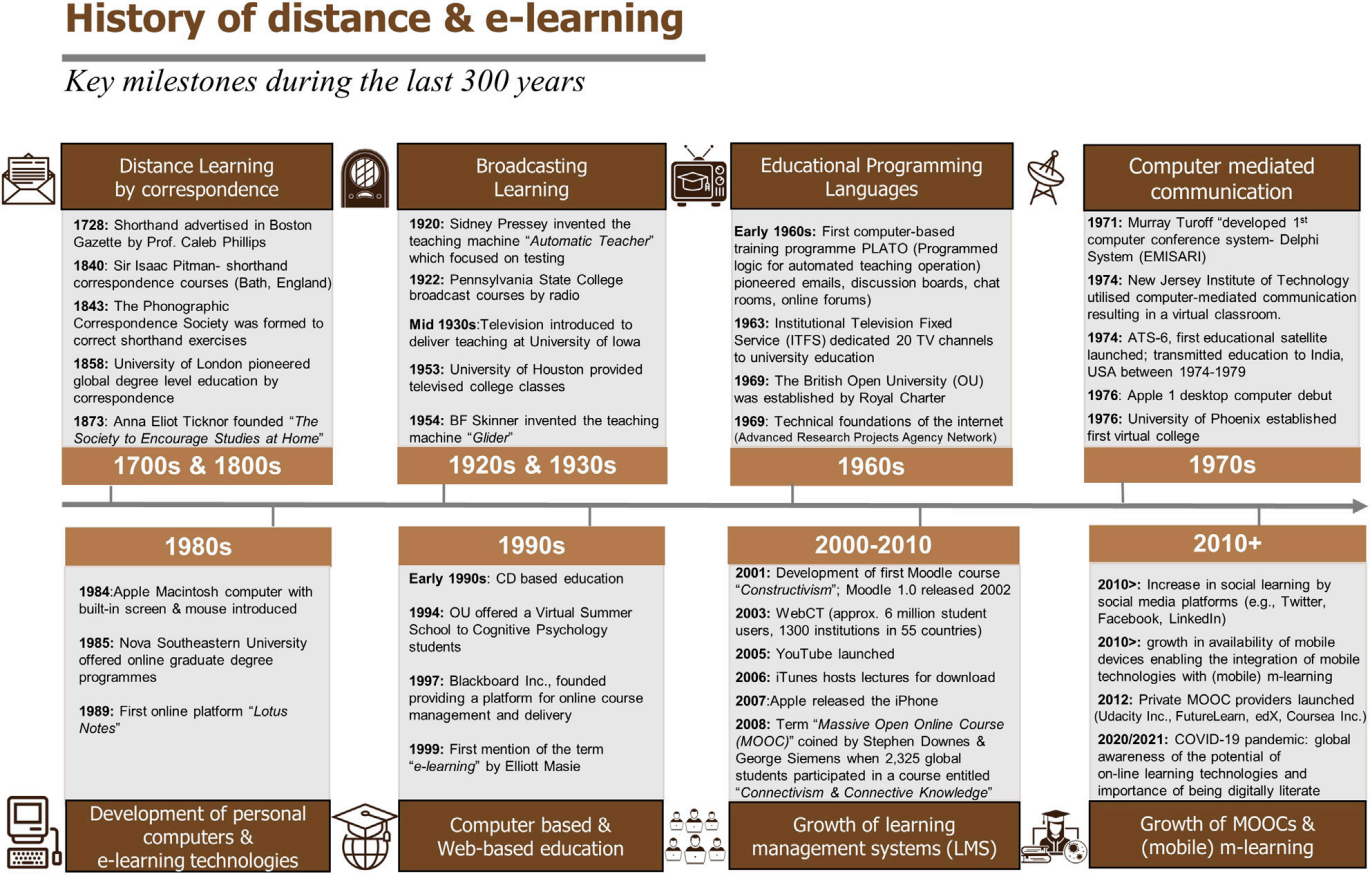
A timeline of key milestones relating distance learning and e-learning. Footer Sources of information used to construct this timeline [6–10].

As technology advanced so did the mode of delivery, the number of education providers globally and the range of educational programmes offered remotely (see Figure 1 for historical milestones in distance and e-learning) [6–10]. The most significant impact was associated with the development of digital computing technologies including the internet and the World Wide Web, which led to online distance learning (e-learning) delivery [1] which has now significantly replaced the previous media of delivery, namely, correspondence, phonograph, radio, telephone and television, particularly in countries with good communication and digital infrastructure [6]. Online platforms and e-learning management systems have transformed the current approach that education providers have taken with the majority offering both blended learning and fully online distance or e-learning education.

**FIGURE 2:**
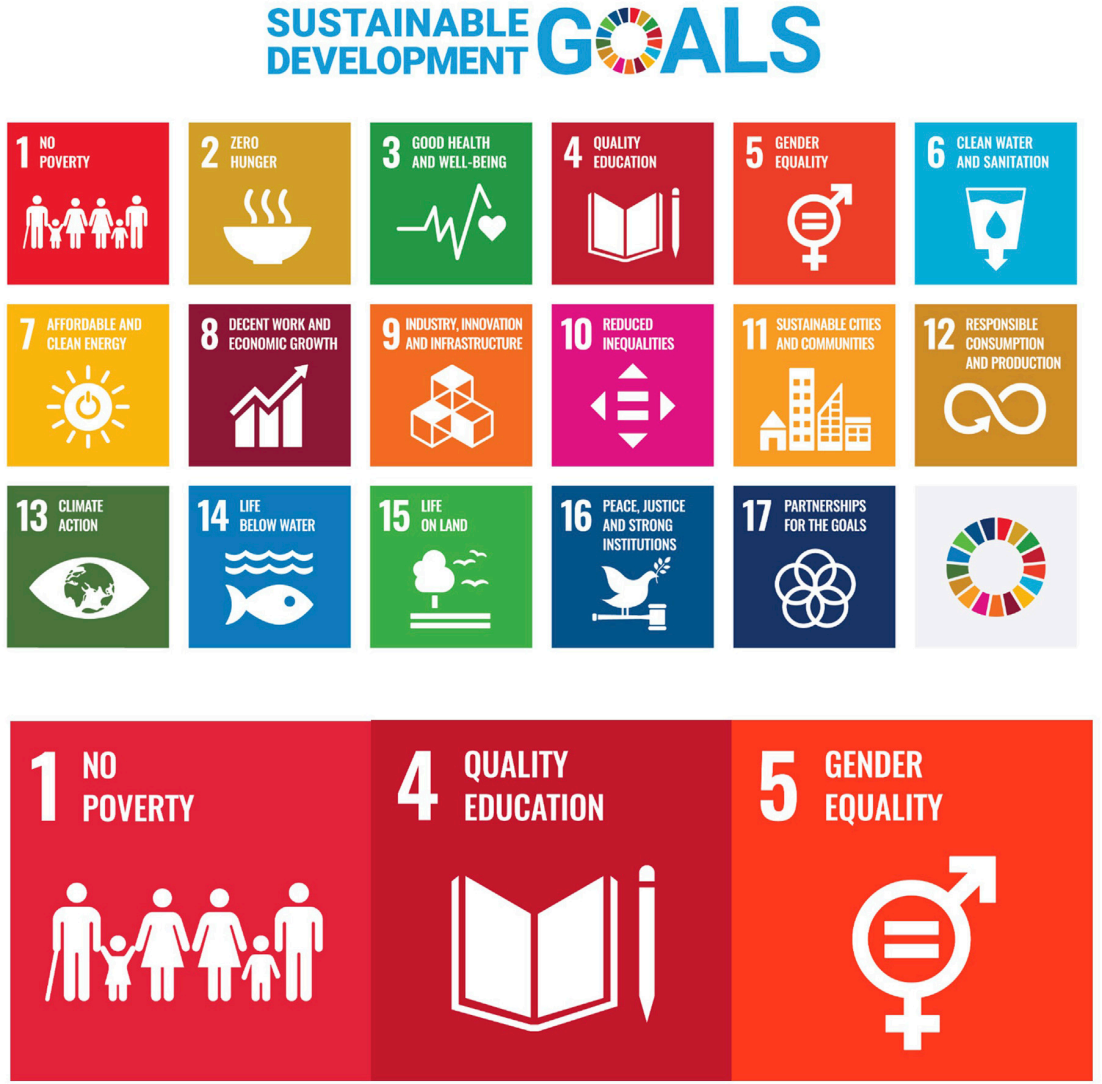
The 17 Sustainable Development Goals (SDGs) adopted by all United Nations Member States [11] and the three primary goals which distance learning makes a substantial input.

Distance learning addresses all 17 UN sustainable development goals (SDGs) (Figure 2), by creating innovative solutions through education, to each of these goals by varying degrees [11], but particularly three of these goals, namely, 1 (No Poverty), 4 (Quality Education) and 5 (Gender Equality) (Figure 2). Access to education has been described as an avenue out of poverty [12]. Distance learning may help alleviate educational costs by staying at home, thus avoiding travel costs to educational institutions, accommodation costs, as well as remaining at home to undertake employment around asynchronous learning platforms. However, poverty can itself impede learning by contributing through a vicious cycle of poor digital access and connectivity within the domestic setting. If digital access can be secured and maintained, e-learners may avail of multiple free web-based resources, but which may not award academic credentials on successful completion. Socio-political factors are important considerations for the introduction and development of distance learning. Komba (2009) concluded that education is an important tool for socio-economic development and a key factor in strengthening human capabilities and reducing poverty in an increasingly globalized world [13]. Distance learning plays a significant contribution to SDG4 (Quality Education). A study from Thailand concluded that for SDG4 to be met, then distance learning requires a quality digital infrastructure of connectivity to allow for sustainable learning [14]. Distance learning may also play a crucial role in helping reduce gender inequality. A study from Tanzania showed that Open and Distance Learning had a crucial role in promoting gender equality and women through empowerment by widening access of education for both women and men and hence improving their socioeconomic and political status [15]. The study showed that the majority of women enrolling did so as it was the only means that they could learn, sustain their career and simultaneously care for their family [15]. A further study from the Philippines made an important point that web-based applications are gender-neutral, which may support the uptake of distance learning [16]. Likewise, Margolis and Fisher 2002 suggested that online education methods are non-sexist and more gender-inclusive [17].

Distance learning programmes are chosen by students for a variety of reasons however it must be acknowledged there are several challenges associated with the delivery of such programmes both for the course team and the student, which need to be addressed (see Table 1). In recent times, the COVID-19 pandemic has forced a root and branch re-examination of provision and capabilities of digital infrastructure and an opportunity to horizon scan to anticipate current and future needs within universities and colleges, as well as within junior, middle and senior schools. It created a watershed relating to the hegemony of physical teaching over distance learning. The barriers and challenges of such “*emergency delivery*” of educational programmes have been extensively reported [18], particularly in relation to the training and final year research project assessments of students enrolled in biomedical science degree programmes [19, 20].

**TABLE 1:** Advantages and challenges of e-learning distance learning educational programmes.

Advantages		Challenges	
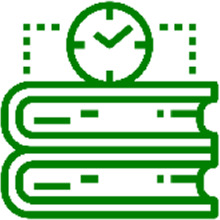	Flexibility	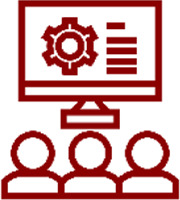	Training requirements
	• Family, work, personal commitments		• Navigation and training relating to online platform
	• Asynchronous life-long learning opportunities		• Ethics, equality, diversity and inclusion and acceptable use policies when online
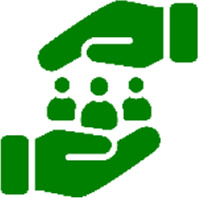	Inclusive	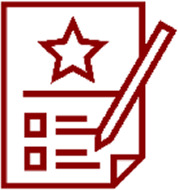	Expectations
	• Available to everyone irrespective of situations/special needs which may not permit traditional in-person requirements		• Support and feedback from tutors ad hoc- realistic expectations need clarified
			• Pastoral care essential
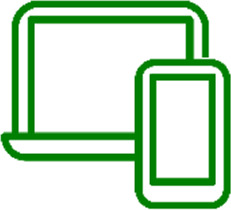	Access	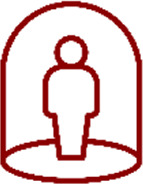	Isolation and engagement
	• Greater access to higher education		• Limited opportunities to engage with peers
	• Multidevice access (phone, tablets, laptop, desktop computers)		• Limited social and peer support • Reluctant to activate microphones and cameras when online
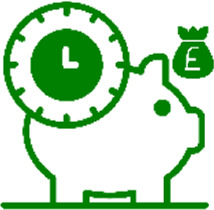	Saving Time and Money	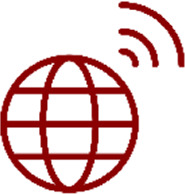	Internet Access
	• No requirement to commute or live on campus		• Poor, limited or no bandwidth
	• Access to online library facilities, e.g., e-textbooks, e-journals		• Access may be limited in institutions such as healthcare settings
			• Technical support outside working hours essential
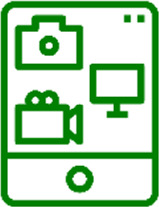	Media Diversity	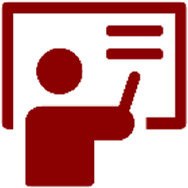	Delivery of teaching materials and resources
	• Text, video, animation, interactive quizzes, discussion forums, webinars, resource sharing platforms		• Interactive teaching and guidance required by tutors
			• On-line formative activities required rather than self-taught
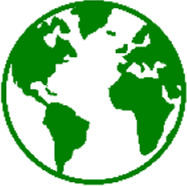	Community learning	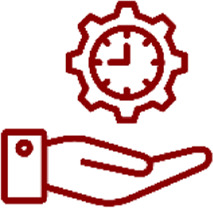	Time-management
	• Global learning opportunities		• Due to work and personal commitments time management is essential to discipline learning and assessment activities
	• Multi-national and multicultural perspectives		
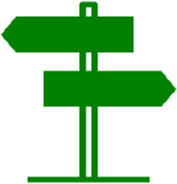	Choice	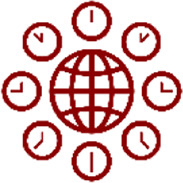	Time difference
	• Wide selection of courses available worldwide		• When online live activities timetabled consideration of student time zones, e.g., record sessions
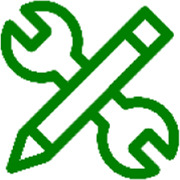	Digital Skills development	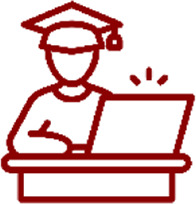	Academic Integrity
	• Opportunities to develop digital competencies during teaching and assessment activities		• Universities require policies and guidelines to support assessment, e.g., guarantors, plagiarism and artificial intelligence checking software

For many individuals, however, online distance learning education has been and still is a personal choice both pre- and post-pandemic, particularly in relation to postgraduate programmes. Within Ulster University, prior to the pandemic, between 2008 and 2019, 4,000 students graduated with an on-line degree/short course qualification from 109 available e-learning courses, supported by 100 part-time e-tutors. More than 70% of these students were enrolled within the Faculty of Life and Health Sciences, indicating the value and interest in distance learning programmes within this discipline area.

Successful completion of the Institute of Biomedical Science (IBMS) accredited MSc degree programmes both on campus and via distance learning, provide eligible IBMS members an opportunity to be recognised as Chartered Scientists [21] and offer a valuable opportunity to promote career progression. As of March 2021, seven universities offer 28 IBMS accredited postgraduate distance learning educational programmes [21]. In relation to the biomedical science postgraduate distance learning courses at Ulster University, enrolment generally comprises of mature allied healthcare professionals who are working fulltime and have various life commitments. As such, distance learning courses offer the required flexibility to enable individuals to balance such commitments whilst furthering their education [22].

It is essential that students enrolled on e-learning programmes are delivered a quality and valuable teaching experience which helps equip them with the knowledge and relevant skills to enable them to be valuable contributors within the workplace and society. The “Know-Do-Be Theory” of education which resulted from UNESCO Commission, “Education for the Twenty-first Century,” is the cornerstone when designing the curriculum and specific learning outcomes and as such direct the key aspects of what needs to be assessed [23].

In the module in this study “Advances in Clinical Microbiology,” it is essential that students, who are generally currently allied healthcare professionals in this discipline specific modality, gain a knowledge of the rapid evolving issues such as antimicrobial resistance, vaccinology and emerging pathogens of global concern, which contribute to “*making the material alive to students*” [24], as well as the development of automated, digital and molecular diagnostic platforms which are transforming the clinical microbiology service. The fourth pillar of “Know-Do-Be Theory” i.e., learning to live together is particularly important in terms of distance learning where students from diverse geographical locations and backgrounds must learn to live and study together and treat others with value and respect [23]. Also, of particular relevance in the twenty-first century holistic model of education is the opportunity to develop competencies and employability skills [25] which is of particular importance in relation to the Integrated Design Framework [26]. Such Biomedical Science online educational programmes at Master’s level also provide students from the biomedical science sector the opportunity to expand and develop competencies aligning with the 2023 updated Health and Care Professions Council (HCPC) standards of proficiency (SOPs), particularly in relation to digital skills and new technologies, leadership, and equality, diversity and inclusion [27]. In addition, the World Economic Forum has detailed the most critical 16 “21st-century skills” required by students to support them as they seek and further their careers in a “technology-mediated world” [28].

A primary disadvantage of e-learning is the minimal opportunities for face-to-face interaction which in turn can impact on students in terms of course satisfaction, engagement, communication, psychology and a lack of sense of community [29]. Distance learning students can often feel isolated due to the temporal separation from both their tutor and peers [22], hence it is important to foster the establishment of online learning communities. The curriculum of the “Advances in Medical Microbiology” module embraces global issues and developments within clinical microbiology and this module provides healthcare professionals with life-long learning opportunities to professionally develop their knowledge of the various emerging issues and developments in clinical microbiology and infectious diseases [30]. This curriculum coupled with an international enrolment, provides a valuable environment for global engagement which should be harnessed so that students can appreciate by learning from each other the real-life impact of the curriculum content from different perspectives across the globe. Interaction of students has been reported as a key element in designing any e-learning activity in general. Various approaches have been used to embed online community interaction and engagement with the e-learning course content, such as collaborative projects, discussion forms and peer evaluation [24]. Although discussion forms are embedded in many online platforms such as Blackboard, learning experiences are more enhanced when a variety of media, i.e., written, visual and verbal, are used to communicate in an online student group [24].

The aims of the development and execution the virtual group assessments described in this paper were to establish an authentic, valid and competency-based group assessment which aligned with the learning outcomes of the module (see Supplementary File S1). This group activity was designed to establish learning communities culminating in the creation of a digital output which would permit the assessment of all six levels of Bloom’s Taxonomy [31]. The development of the creative outputs and their subsequent sharing and evaluation by peers coupled with the consequential validity of the assessment on student learning, would provide students the opportunity to expand microbiology discipline specific knowledge. Additionally, this would promote the development of transferable digital, reflective and social competencies, such as communication, collaboration leadership and respect of others [32].

## Materials and Methods

### Assessment Design

Two group-based e-learning assessments constructively aligned with the learning outcomes of a discipline specific 30 credit point module (BMS 858- Advances in Medical Microbiology; Supplementary File S1) in part fulfilment of an IBMS accredited distance learning MSc programme in Biomedical Science. Assignments were constructed to promote and enable 1) the establishment of online learning communities; 2) development of specialism-specific knowledge relating to clinical microbiology and 3) development of transferable skills including critical thinking, communication, collaborative team and digital skills. Throughout the group activities students were provided with e-tutor support to help direct the students to reliable resources and practical guidance on the use of the various digital platforms and tools which were required for these activities.

#### Group Activity 1

The first group activity was conducted during November/December 2019, i.e., prior to the COVID-19 pandemic. Twenty students enrolled in this module. The group assignment focused on viral pathogens. Each group of four students was given a named virus which was of current clinical interest due to several factors including epidemiology, clinical significance, vaccine properties and availability. Each group was given a specific aspect/problem to focus on and was asked to critically examine this aspect in light of current peer-reviewed research and global policies established by respected institutions, e.g., WHO, Government Bodies. The topics and assignment components are detailed in Table 2.

**TABLE 2:** Group assignment topics and activities.

Assignment 1: Slide Deck with slide notes (presentation using Blackboard Live Sessions optional).	
Group	Topics
1	Ebola virus: *“An ongoing challenge to prevent further global outbreaks”*
2	Measles morbillivirus: *“Tackling Re-emergence: vaccine failure or vaccine phobia?”*
3	Human papillomavirus: *“A vaccine necessary for all sexes”*
4	Human immunodeficiency virus: *“An HIV vaccine- is the end in insight or are there still barriers to overcome?”*
5	Poliovirus: *“Successful elimination or concern: emergence of vaccine-derived polioviruses”*
**Mark allocation**	**Activities**
10% (Group mark)	Strategy on a page
30% (Group mark)	Preparation of a Slide Deck (20 slides)
10% (Group mark)	Preparation of a Reference List
10% (Personal mark)	Personal slide content and preparation
20% (Personal mark)	Assessed Group Discussion Board
10% (Personal mark)	Written reflection
10% (Personal mark)	Audio-visual reflection shared with module members

Assignment 2: Debate Presentation using voiceover PowerPoint, Infographic, Voting.	
Group	Topics
1	*“Antimicrobial resistance is of a greater global concern than emerging/re-emerging pathogens.”*
2	*“Automation will result in a better service with manual approaches within diagnostic clinical microbiology becoming obsolete.”*
3	*“The availability of recent novel meningococcal vaccines will result in the eradication of meningococcal disease.”*
4	*“Molecular diagnostics within clinical microbiology is superior to culture-based approaches which will become redundant.”*
5	*“Prevention is better than cure- vaccines rather than novel antimicrobials are the future for combating infectious diseases.”*
**Mark allocation**	**Activities**
Formative	Strategy on a page
40% (Group mark)	Debate presentation and Summary Infographic
20% (Group mark)	Robust and current sources of information, accurate referencing of slides/list
10% (Personal mark)	Peer-mark
10% (Personal mark)	Self-mark
20% (Personal mark)	Assessment and voting on other group debates with justification
Separate assignment	Written reflection and audio-visual reflection (Completed at end of module after completion of all assignments)

For assessment purposes, students were requested to collectively prepare a slide deck, slide notes and accompanying reference list relating to their assigned topic. Initially, groups prepared a strategy on a page of how the group was going to approach the assignment, detailing how and when they would meet, the various responsibilities of the members of the group, sources of information and internal group deadlines. Following feedback from their e-tutor, the groups executed their strategy plans. The final slide deck was shared with all members of the module to enhance their knowledge of the five subject areas and provided a further source of information to help prepare students for the end of modular examinations. Students were not required to prepare a voice over presentation, however two groups wished to deliver a live oral presentation via Blackboard Ultra, to their e-tutor on completion. All students were also asked to communicate with their group members using assessed group discussion forums throughout the preparation stages. Having completed the group task, students were asked to personally reflect using written format and prepare an audio-visual reflection to share with their peers. Audio visual reflections were recorded using the digital tool FlipGrid [33].

#### Group Activity 2

The second group activity was conducted during November/December 2020, i.e., during the COVID-19 pandemic and following analyses of the previous academic year’s activity. Twenty-one students enrolled in this module. The group assignment focused on global topical and debatable issues in clinical microbiology. The topics and assignment components are detailed in Table 2. This form of group assignment task was to encourage students to expand their critical evaluation skills rather than solely presenting fundamental knowledge of specified subject areas. The implementation of further digital skills were embedded to enhance students’ digital communication skills by including both verbal and visual formats to prepare a digital output which was engaging, critical, informative and concise.

In this assignment task, students were placed into groups of four or five and were asked to retrieve and critically analyse scientific literature to evaluate the assigned topic. A strategy on a page was prepared and the e-tutor provided feedback as was the case in assignment 1. The completed assignment output took the form of an online debate and groups were required to create a PowerPoint recorded audio-visual presentation of the debate together with a summary infographic and reference list. Subsequently, all debate presentations and infographics were uploaded to the module portal and students were asked to examine these and vote on which side of each of the motions they supported, also providing a justification. When all group work was completed, each member of the group provided a peer-mark and self-mark in relation to the contribution of individuals during the group task (see Supplementary File S2 for the peer-/self-marking template). At the end of the module, students were asked to personally reflect using written format and prepare an audio-visual reflection to share with their peers. Audio visual reflections were recorded using the digital tool FlipGrid.

### Evaluation Methodology

This ethically approved study [Ulster University procedures for research involving human subjects (FCBMS-19-091)] comprised of a mixed methods approach to evaluate the introduction of virtual group assessments. Qualitative data was gathered through reflective feedback and was used to evaluate the assessment outcomes in terms of experiences, skills development and an appreciation of employability skills. Quantitative data and statistical evaluation allowed further refining and evaluation of the outcomes of this project from data gathered through questionnaires and a content analysis of the reflective outputs [34].

### Surveys

Prior to commencing group activities and subsequently following completion of these activities, all students enrolled in the module were invited to complete a voluntary pre- and post-skills evaluation, which comprised of a 5-point Likert Scale questionnaire (Supplementary Files S3, S4). Responses from students who provided written consent were analysed. For statistical analyses, qualitative Likert responses were assigned values 1–5 as detailed in the survey questionnaires (Supplementary Files S3, S4). The pre- and post-questionnaires provided students the opportunity to quantitatively evaluate their skill development during their undergraduate degree, and assignment tasks in relation to problem solving, critical thinking, creativity, group coordination, preparation of digital media and digital communication. The questionnaires also provided students the opportunity to quantitatively assess the importance of transferable skills development in relation to securing employment and career progression within the biomedical science sector.

### Qualitative Data Content Analysis

A content analysis was performed on the reflective writing of each student [35]. Categories were constructed focusing on pre-assignment apprehensions, challenges encountered, value of group activities, skills developed and personal feelings and attributes on completion of the final group output.

### Statistical Analyses

Statistical analyses were performed using non-parametric methods. For all data, a Kolmogorov-Smirnov test for normality was conducted prior to a Kruskal-Wallis test and *post hoc* by Dunn’s multiple comparisons test for related groups which were not normally distributed. Statistical significance was set at *p* ≤ 0.05. Chi-square and Fisher’s Exact tests were also conducted. All analyses were conducted using GraphPad Prism 10 for Windows, Version 10.0.0 (GraphPad Software, Boston, United States).

## Results

### Surveys

The uptake rate of the pre- and post-group assignment surveys for the Group Activity 1 was 80% (16/20) and 52% (11/21) respectively. The uptake rate for the pre- and post-assignment surveys for the Group Activity 2 was 38% (8/21) and 33% (7/21), respectively.

**FIGURE 3:**
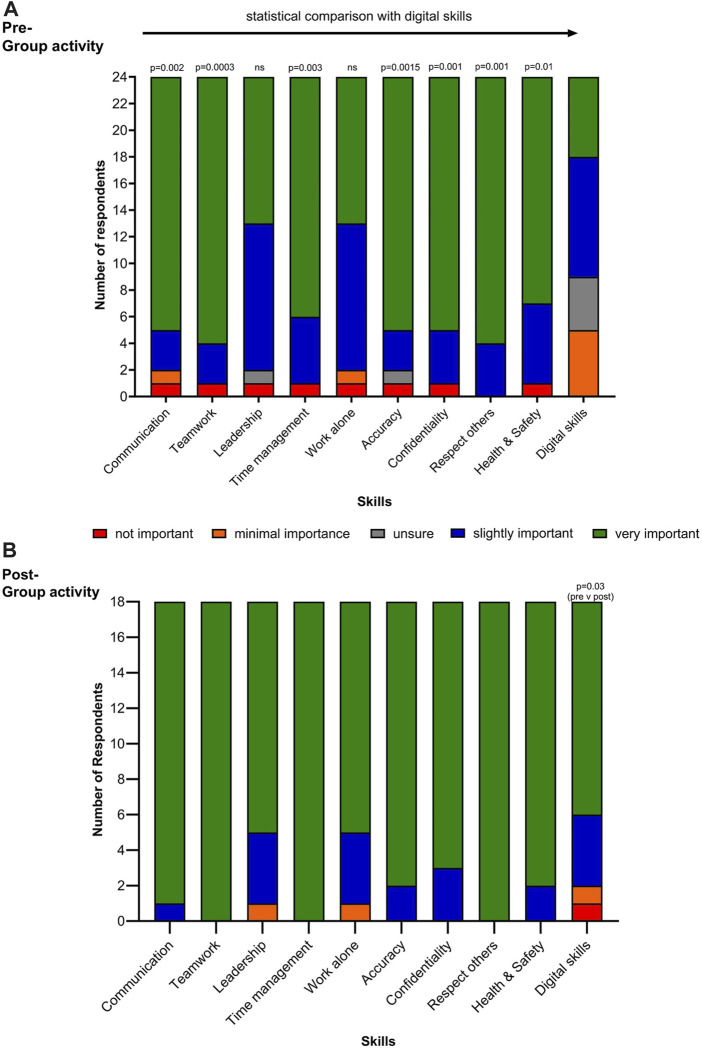
Respondents evaluation of the importance of developing work-related skills during postgraduate study to help secure a job in the biomedical science sector: **(A)** Pre-Group assignment activities (*n* = 24; statistical significance is noted between digital skills and other transferable skills) and **(B)** Post-Group assignment activities (*n* = 18; statistical significance was only noted between pre- and post-Group assignment activities in relation to digital skills).

Responses (*n* = 24) to the pre- and post-questionnaire in relation to the importance of developing work-related skills during postgraduate study to help secure a job in the biomedical science sector is shown in Figures 3A, B, respectively. In relation to the pre-assignment survey, there was a significant difference in responses indicating a lower importance being attributed to digital skills development than skills development with respect to communication (*p* = 0.002), teamwork (0 = 0.0003), accuracy (*p* = 0.0015), confidentiality (0.001), respect for others (*p* = 0.001) and health and safety (*p* = 0.01).

No significant difference was evident in the skills which respondents believed were essential to develop during postgraduate study in relation to job acquisition versus job progression (Supplementary File S5).

In relation to the post-assignment survey (*n* = 18), there was a significant difference in responses in relation to an increased importance being attributed to digital skills development (*p* = 0.03) than indicated in the pre-assignment responses. Although there was a trend towards an increased importance, to the development of other skills, these were not significantly different.

**FIGURE 4:**
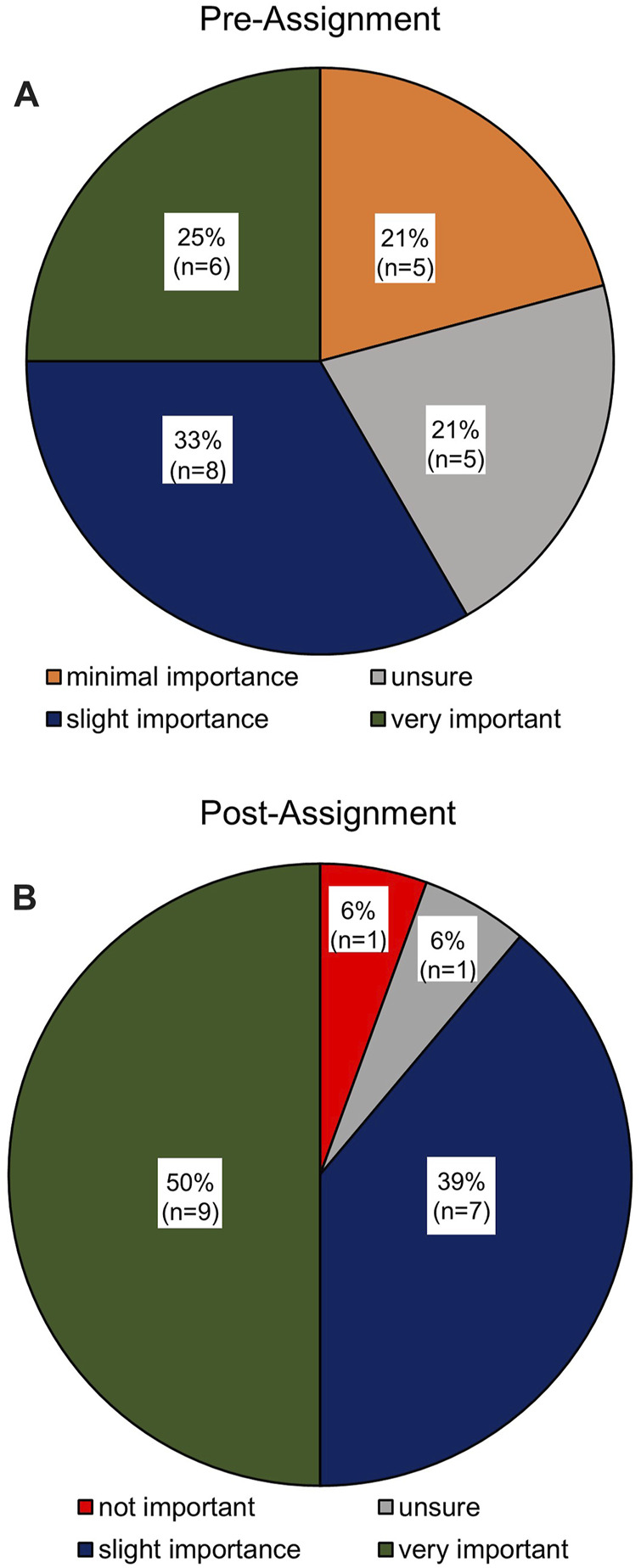
Respondents evaluation of the importance of digital skills to employers within the biomedical science sector. **(A)** Pre-Group assignment activities (*n* = 24). **(B)** Post-Group assignment activities (*n* = 18). A significant increase in importance (*p* = 0.04) placed on relevance to digital skills following the completion of the assignment tasks.

Figures 4A, B, pre- and post-assignment, respectively, demonstrate a significant increase in importance (*p* = 0.04) on the relevance of digital skills to prospective employers, following the completion of the assignment tasks.

**FIGURE 5:**
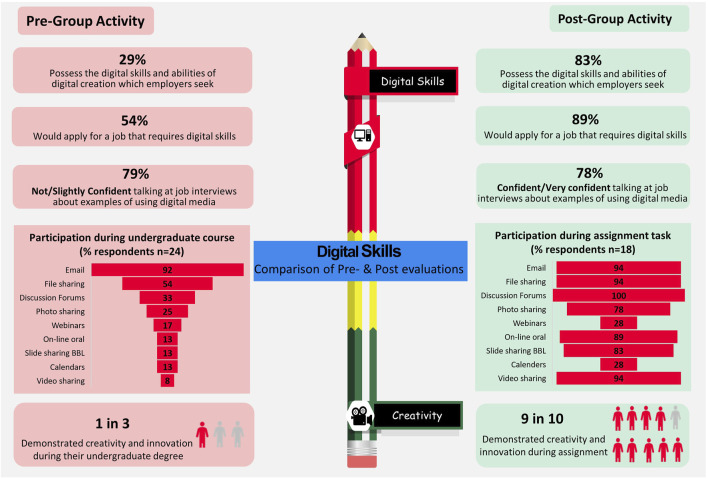
Infographic of a comparison of key areas which improved following students completing the group assignment activities.

The infographic shown in Figure 5 summarises respondents pre- and post-assignment responses and indicates that following completion of group tasks, there was an increase in respondents indicating that they had the digital skills and abilities of digital creation which employers seek and that more students would apply for a job that requires digital capabilities. An increased range of digital platforms and digital tools were utilised during the group assignment activities with an increase in the number of respondents who indicated they had demonstrated creativity and innovation during the group task compared to during their undergraduate degree.

**FIGURE 6:**
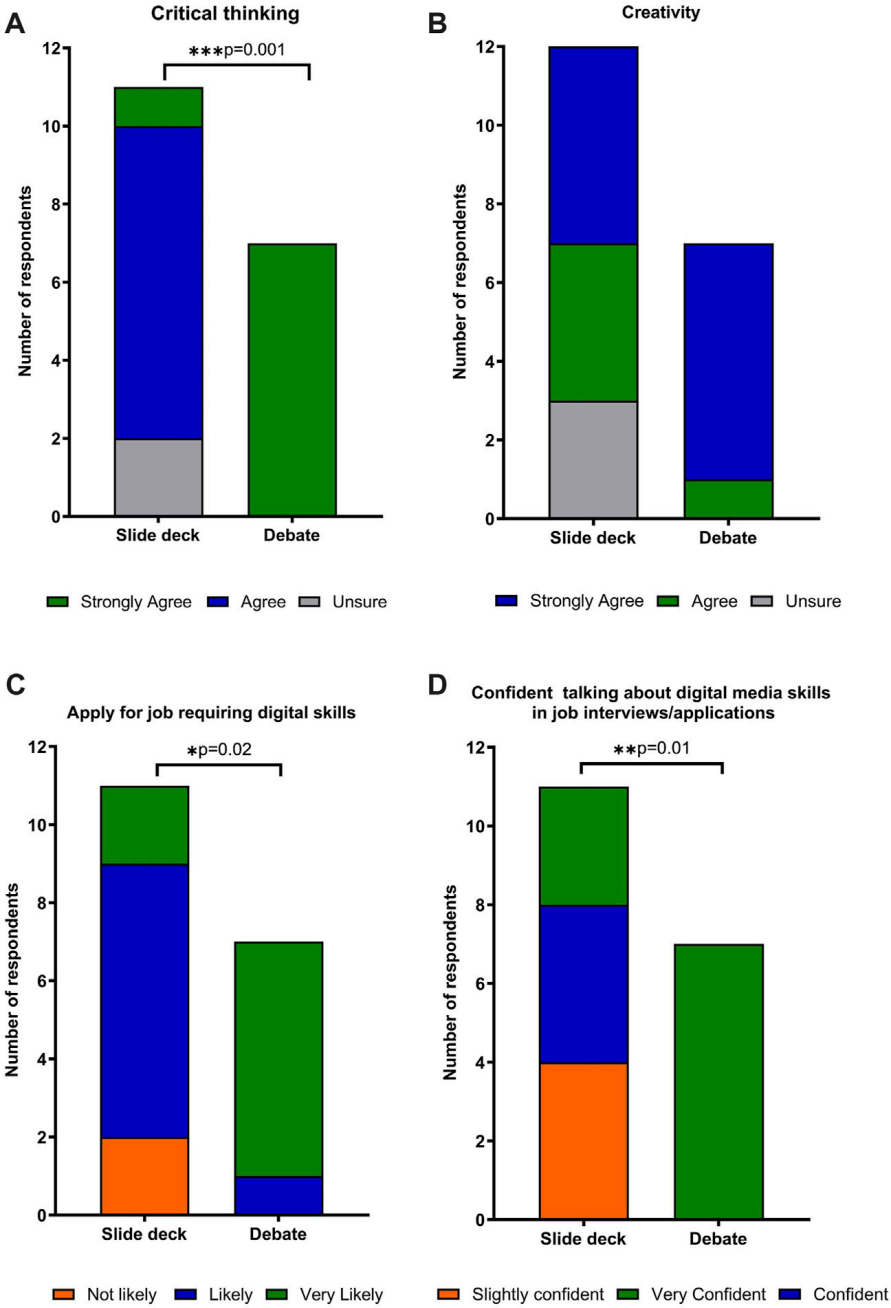
A comparison of respondents’ views after completion of the two group assignment activities in relation to **(A)** demonstrating their critical thinking skills; **(B)** demonstrating their creative skills; **(C)** likelihood of applying for a job requiring digital skills and **(D)** confidence in talking about examples of using a range of digital media in job applications and interviews.

From the responses to post-group assignment questionnaires in relation to a comparison between two group activities, it was shown that there was a statistical increase in the proportion of respondents (*p* = 0.001) who strongly agreed that they developed their critical thinking skills during the debate activity in comparison to the slide deck activity (Figure 6A), with no statistical difference in relation to creativity (Figure 6B). In comparison with the slide deck activity, following completion of the debate activity, more respondents indicated that they would apply for a job requiring digital skills (*p* = 0.02) and that they would be confident in talking about examples of a range of digital media skills during job applications and interviews (*p* = 0.01) (Figures 6C, D).

### Qualitative Data Content Analysis

**FIGURE 7:**
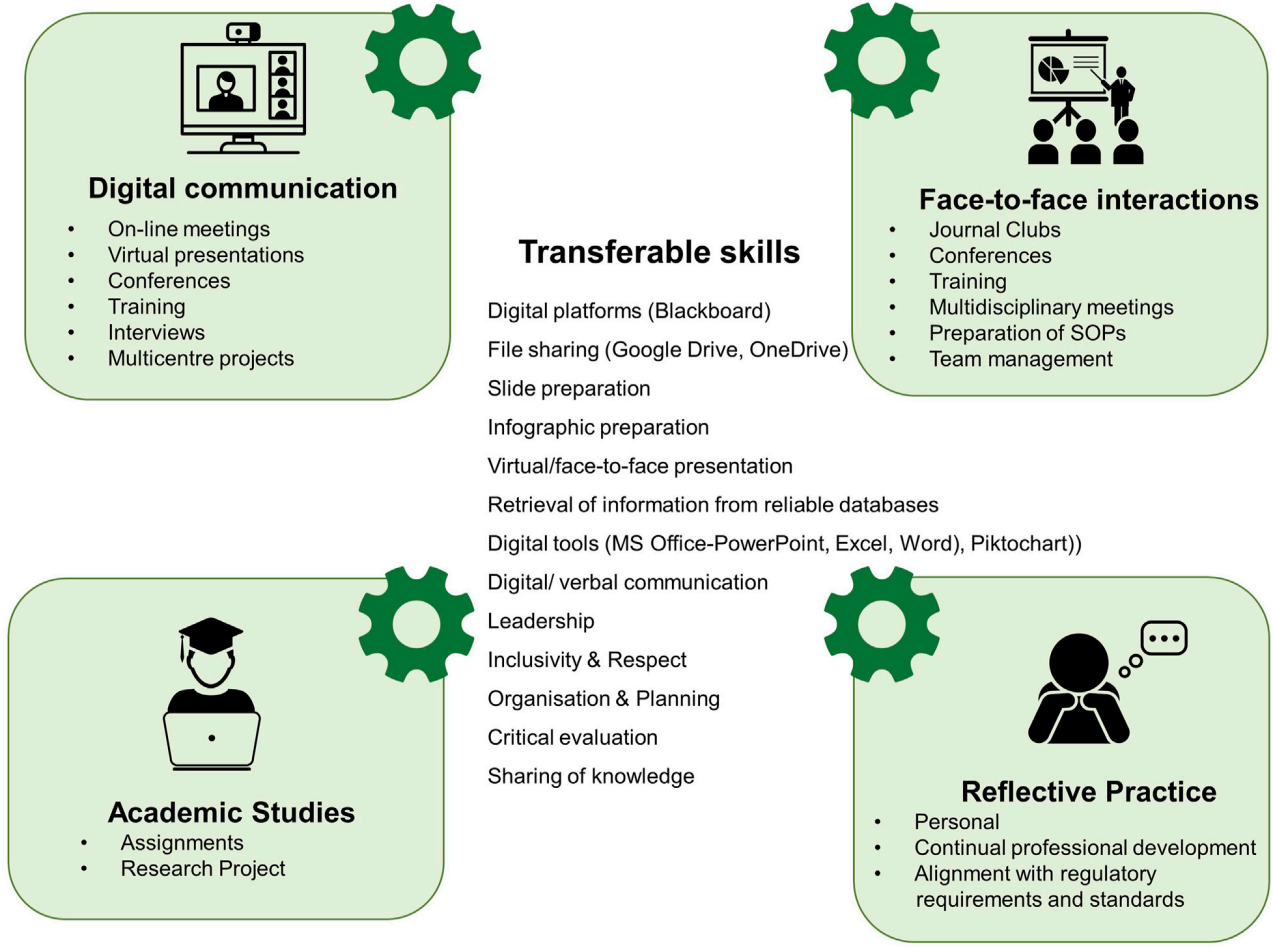
Areas where students detailed they would use the skills and knowledge gained during the group assignment activities.

Following a content analysis of students’ reflections [Group Activity 1 (*n* = 20); Group Activity 2 (*n* = 21)] a statistical comparison of common themes was conducted using a Fisher’s exact test with Odds ratio and is shown in Table 3. In relation to statistically significant differences between the two assignment tasks, there was a higher proportion of students who completed the debate activity who initially expressed concern about a group activity (*p* = 0.001) and indicated difficulties with time commitments to meet (*p* = 0.004). Within the debate cohort a greater proportion reported developing leadership (*p* = 0.045) and critical evaluation skills (*p* = 0.045). In the Slide Deck cohort, there were statistically more students who reflected on their collaboration/teamwork skills (*p* = 0.028). All students reflected that their initial pre-assignment concerns were alleviated during completion of the group tasks with approximately 70% of students reflecting on the collegiality during the group activities. Most students acknowledged the knowledge gained (85%, 86%) and the skills developed in relation to digital platforms and digital tools (80%, 90%) during the group assignment activities, slide deck preparation and debate, respectively. Students reported that the group activities were an enjoyable experience in relation to the slide deck preparation (70%) and debate (67%). In the case of the debate activity, 62% of students reported that they had developed confidence in performing future tasks both in academia and education which would require the skills they had developed (Table 3; Figure 7).

**TABLE 3:** Quantitative analysis of students’ reflections following group assignment tasks.

	Slide deck (Total = 20) % (*n*)	Debate (Total = 21) % (*n*)
Pre-assignment apprehensions		
• Logistics and difficulties of working as a group online	35 (7)	62 (13)
• Working with unknown individuals	35 (7)	19 (4)
• Group activity	—	43 (9)^a^
• Not all group members would participate equally	30 (6)	29 (6)
• Unfamiliar with digital platforms/tools	30 (6)	24 (5)
• Debate	—	19 (4)
• Reliability of internet	5 (1)	—
• Timely feedback from others	5 (1)	—
• Unsure where to start	5 (1)	5 (1)
• Prefer to work alone	5 (1)	10 (2)
• Co-dependence to achieve assessment mark	5 (1)	5 (1)
• Volume of work	—	10 (2)
• Different time zones	—	19 (4)
Challenges		
• Time to meet as a group due to other commitments	65 (13)	19 (4)^b^
• Time management	25 (5)	48 (10)
• Unfamiliar with digital platforms/tools	30 (6)	24 (5)
• Discussion boards- responses not in real time	20 (4)	—
• Responsibility- not wishing to let the group down	20 (4)	5 (1)
• Apprehensive regarding audiovisual recording (FlipGrid)	10 (2)	5 (1)
• Reluctant to turn on camera when communicating remotely	10 (2)	—
• Limited public speaking/presentation skills	—	14 (3)
• Lack of creative thinking skills	—	5 (1)
Value of the group activity		
• Knowledge gained	85 (17)	86 (18)
• Enjoyable experience	70 (14)	67 (14)
• Collegiality	70 (14)	71 (15)
• Interesting	65 (13)	52 (11)
• Peer interaction and support in general	50 (10)	67 (14)
• Importance of inclusivity and others’ viewpoints/abilities	45 (9)	52 (11)
• Continual professional development	25 (5)	38 (8)
• Learnt from others	10 (2)	24 (5)
• Relevant topics	15 (3)	10 (2)
Skills developed		
• Digital platforms/tools	80 (16)	90 (19)
• Communication	75 (15)	52 (11)
• Collaboration/Teamwork	75 (15)	38 (8)^c^
• Time management	50 (10)	62 (13)
• PowerPoint	45 (9)	62 (13)
• Organisational skills	25 (5)	29 (6)
• Reflective Practice	20 (4)	38 (8)
• Using literature search tools	15 (3)	10 (2)
• Leadership	5 (1)	33 (7)^d^
• Critical evaluation	5 (1)	33 (7)^e^
• Presentation/public speaking	—	38 (8)
• Flexibility	—	14 (3)
• Problem solving	—	14 (3)
• Creative thinking	—	5 (1)
• Decision making	—	10 (2)
Personal feelings/attributes		
• Confidence	35 (7)	62 (13)
• Proud of achievement/final product	30 (6)	43 (9)

Statistical significance as per Fisher’s exact test.

^a^ *p* = 0.001 (Odds ratio 0.000; 95% CI 0.000–0.316).

^b^ *p* = 0.004 (Odds ratio 7.893; 95% CI 1.973–26.78).

^c^ *p* = 0.028 (Odds ratio 4.875; 95% CI 1.355–18.84).

^d^ *p* = 0.045 (Odds ratio 0.105; 95% CI 0.009–0.778).

^e^ *p* = 0.045 (Odds ratio 0.105; 95% CI 0.009–0.778).

## Discussion

Online group assessment, requires extensive planning, assignment design and access to student support to ensure effective strategies are in place to counteract any challenges faced by academic staff or students when undertaking such activities [36]. This form of assessment was considered in this current study, as it ultimately fosters student negotiations in relation to co-construction and constructive conflict, leading to a mutual cognition resulting in the development of deeper learning and interpersonal skills, all of which are relevant within professional practice [37]. Furthermore, a group-based activity helps to establish an on-line learning community promoting “*team engagement*” and “*collaborative creativity*” [38], which encourages an “*interaction-knowledge network*” and a sociocognitive approach to learning by aligning to real-life collaborative scenarios.

In the case of the current group assignments, students acknowledged the opportunity to execute their creative skills (Figure 6B) and the group activities fostered good teamwork, building a collegiality which resulted in successful attainment of the assessment learning objectives and the construction of a final product which students felt proud of (Table 3). The pinnacle of Bloom’s modified taxonomy is creativity, which these group activities promoted, however, it has been reported that students of biomedical science may not realise creative opportunities [39]. Educators, therefore, have a responsibility to define creativity and innovation; particularly where this applies to the learning outcomes through assessment, enabling students to recognise and harness their creative abilities and appreciate the value of these skills in terms of employability [39]. Educators must also be mindful when designing group activities, that dominant members do not negatively impact on the activity of their peers [38]. From the student reflection in relation to these assessments, this did not however appear to be an issue, in contrast students reflected they were encouraged and supported by their peers and felt great pride for their co-produced digital output (Table 3).

The challenges which have presented in the integration of online groupwork need to be acknowledged and have been discussed in a recent systematic review by Donelan and Kear [36]. Several of these challenges were identified during the two group assignment activities, namely, i) initially prior to the commencement of the group activities, students were concerned that not all group members would participate equally, however such fears were rapidly dispelled once the members of the group made initial contact; ii) scheduling and time issues impacted on the ability to hold virtual meetings in real time and iii) anxiety was reported in relation to having to work with strangers in a remote situation (Table 3). Students overwhelmingly however, valued the group assessments and many reported that these activities promoted them to take on leadership roles, both within the assessment and subsequent translation within the workplace.

**FIGURE 8:**
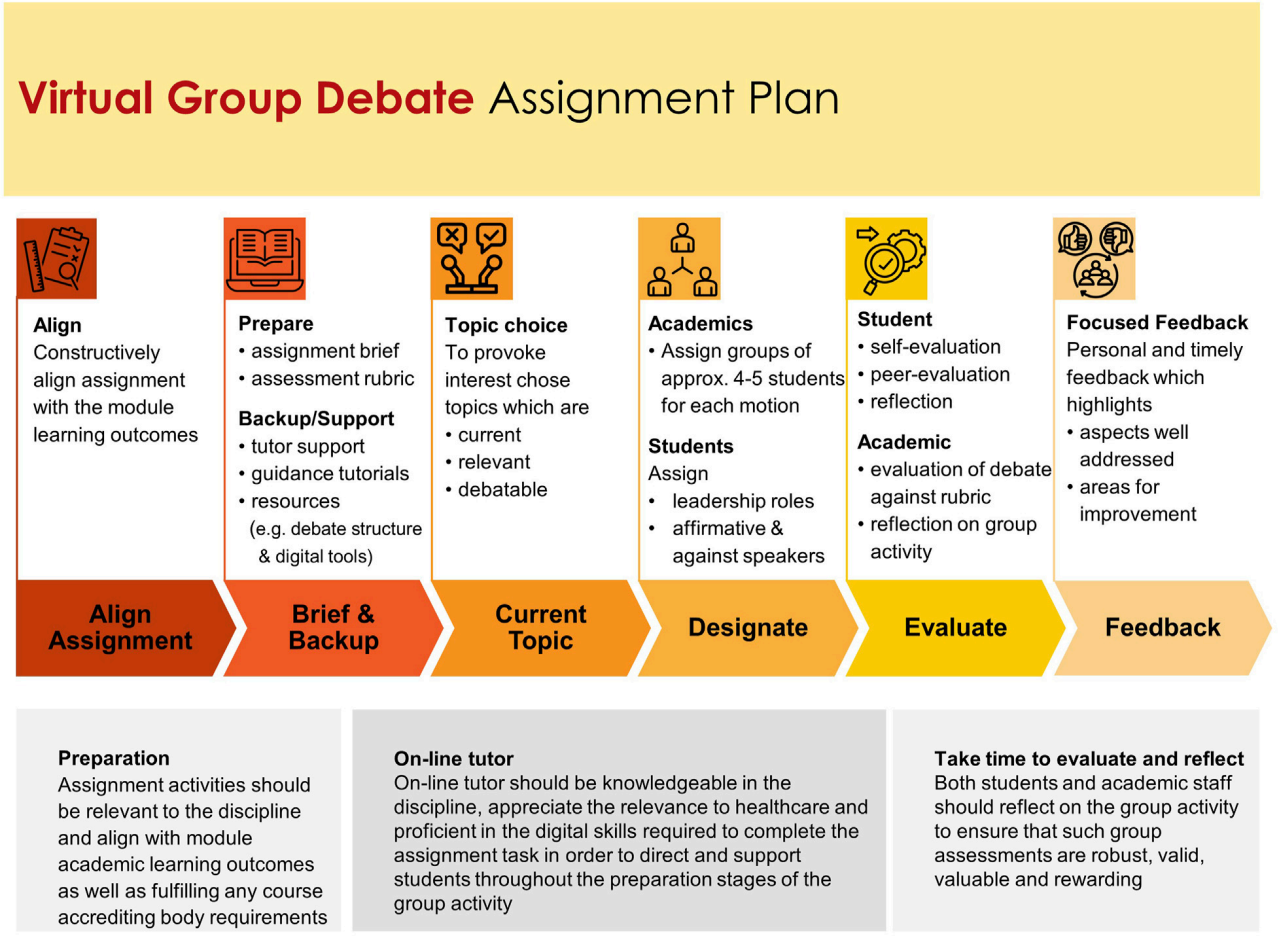
Virtual group debate assessment plan.

Donelan and Kear [36], concluded that although the literature identifies numerous challenges, for which strategies have been developed to address, there are two fundamental areas which are a priority for consideration, namely, i) that initially all aspects of the group activity are carefully designed supplying students with detailed guidance and preparation and ii) the groups’ relationships are supported throughout the duration of the activity [36]. It was therefore essential, academics robustly developed an assignment plan (Figure 8) and that early in the module clarity was provided to students in relation to i) which aspects of the group activity were being assessed, i.e., individual/group contribution, ii) details regarding the process and tools used to prepare the final product and that e-tutor support was available throughout the process to offer guidance. Such clarity helped to alleviate any concerns students may have had relating to the assignment task. This approach is also transferable to online group debates in other healthcare and non-healthcare educational programmes.

To guide developments relating to assessment, it is important to critically appraise evidence-based practice approaches and assessment guidelines in conjunction with the students’ voice, gathered by means of local student feedback and reflections to help mould the final product into an engaging, challenging, criterion-referenced assessment. Such assessment embeds the translation of relevant pedagogical theories; particularly those of Anderson and Krathwohl’s modification of Bloom’s Taxonomy [40] and Biggs’ constructive alignment [41], into educational practice, to help students become effective learners. Following the assessment of the first group activity in which student cohorts prepared a slide deck on topical issues in clinical microbiology, it was considered by the course team that although the students enjoyed the activities and their knowledge of the subject area was enhanced, a more critical appraisal of the topics could have been promoted. Additionally, in relation to the group 1 assessment activity, two groups were proud of their achievements and although not compulsory, wished to orally deliver their slide deck via a live Blackboard Ultra session. As such, the group assignment was reconstructed to include a recorded oral presentation in a debate style format to ensure a positive student experience focusing on higher order thinking processes and competencies, as well as summative and formative learning approaches to help prepare students for the workplace environment [25, 32].

Debate incorporates constructivist pedagogies and offers a platform for students to express and consider diverse thoughts and ideas [42]. This activity has been used effectively in face-to-face teaching to develop and assess higher-order cognitive and communication skills, stimulating interest and confidence and acquire knowledge in microbiology [42–44]. On-line debate encourages group interaction in an innovative and a more engaging manner using digital communication and presentation rather than the use of traditional discussion forums [45]. Indeed, students reflected that they found the use of discussion forms frustrating as these forums did not allow communication in real-time (Table 3). If conducted asynchronously, on-line debate could permit a more critical evaluation of the scientific evidence before constructing a response, thereby providing a pedagogical strategy promoting reflexivity [46]. On-line debates have not been used extensively as an assessment model which may be due to the fact that their coordination and evaluation requires significant input particularly if delivered via Blackboard Discussion Forums [47], as such it was decided that the delivery of these online debates would be by means of an audio-visual format, namely, voice over PowerPoint. Careful consideration was required in relation to the logistics and digital tools required within the online setting. With provision of the appropriate digital platforms and tools and e-tutor guidance, students successfully delivered their voice over debates, which resulted in an improvement in utilising critical thinking skills in comparison with the preparation slide deck in the first group activity (Figure 6A).

The challenge associated with developing assessment is to ensure it is effective and fair to all students and staff, as recently considered within JISC’s report [48], “*The future of assessment: five principles, five targets for 2025.*” This report examined how new technologies, namely, digital tools and platforms, can improve and ensure assessment is authentic, accessible, automated, continuous and secure, resulting in students having a fulfilling learning experience, as they develop skills and character during their education and for future employment. Online group activities offer an ideal environment for students to develop their leadership, time-management, collaborative and organisational skills but most importantly digital communication and digital creation skills all of which are central to the success of online group activities as well as a skill set transferable to the workplace [36].

While this study has focused on the evaluation of the development of microbiological knowledge and transferable skill development, when embedding a new form of assessment, it is of utmost importance to provide students with feedback. Feedback and more effectively feedforward coupled with clear instruction is an important aspect of assessment and the meta-cognitive experience [49, 50] contributing to students becoming effective learners by correction and development. The initial submission of a group strategy, enabled students to receive feedback which they could then subsequently feedforward into the development of their final output.

Such feedback guides students to understand requirements in terms of their understanding and demonstration of knowledge and promotes development through reflection, thinking, reading and writing [51]. In the case of distance learning students, feedback is a source of intrinsic motivation [52]. Consideration is required when delivering feedback in relation to a group activity to ensure all students fully appreciate and value the feedback in terms of both their own performance and in relation to group performance. Feedback in the form of peer assessment provides an opportunity to challenge students cognitively and highlight areas which deviate from the standards required [53] and should be considered in group-based assessment. A number of feedback modalities both written and verbal may be used to support students’ varied needs and preferences [49]. Students report a preference for written feedback [54], although verbal and face-to-face feedback are considered more personal and thorough [55]. In the case of these assignments, the e-tutor delivered personal written feedback and general audio-visual feedback to the complete cohort of students. Overall, whether feedback is written or verbal, it is important to ensure the tone and language used is not derogatory but offers valid, balanced, personal and motivational advice linked to learning outcomes which students can respond to in order to promote improvement [56].

Reflection is of fundamental importance to all allied healthcare professionals and is central to their continual professional development. Many education providers of biomedical science programmes have embedded reflection into their assessments [57, 58]. Reflection can take many forms, visual, written, audio, etc. and valuable resources are available for healthcare professionals and academics to promote to their students [59–61]. Within these assessment tasks, two modalities of reflection were incorporated, namely, written and a reflective video. The reflective video was prepared using the free Microsoft app FlipGrid (now known as Flip), a social learning community app, which educators globally have used to create safe online groups for students to communicate and convey their thoughts and ideas using short video messages [33]. FlipGrid has been used in all levels of education to promote student engagement and discussion [62]. In relation to higher education, FlipGrid has been used to promote scientific communication and enhance oral skills [63]. It has been used both by students and academics and has the potential to help build online educational communities [63]. FlipGrid has also been used to facilitate and improve critical reflection by collaborative online interactions in a undergraduate Sports Coaching degree programme [64].

Students in this study who used the FlipGrid to reflect reported that this digital tool was easy to use and that they enjoyed being able to see that they were part of a larger group of individuals who had the same successes, challenges and reflective thoughts as their peers, with only two students (2/41) indicating that they found the recording of a reflective video challenging and stressful. It should be noted however, that FlipGrid also provides the opportunity to make audio posts and students can use a still image or avatar image if they do not wish to reveal their face [65]. Further studies are required to fully examine the potential of utilising FlipGrid to promote e-learning communities and asynchronous interactive discussions in the discipline of biomedical science.

In addition to the knowledge acquisition gained during the completion of these two group activities, it was evident that the skills which students developed were of particular value in relation to digital platforms, digital tools and digital communication. These are important skills to develop, particularly in relation to the updated HCPC Standards of Proficiency (SOPs), effective from 1 September 2023, which have emphasised the need to be able to keep up to date with digital skills and new technologies, namely, 7.7, which addresses effective communication “*Registrants must use information, communication and digital technologies appropriate to their practice*” [27]. Both of these group-based activities were conducted prior to the updating of the SOPs and it is interesting to note that prior to the assessment tasks students attributed a lower importance to the development of digital skills than skills development with respect to communication, teamwork, accuracy confidentiality, respect for others and health and safety. This highlights the importance of embedding and highlighting where such digital skills are useful within the healthcare setting and the biomedical science sector [66]. Within this study, students reflected and acknowledged areas where they had already used or would potentially use such digital skills in their workplace (Figure 7). It must also be acknowledged that the majority of students enrolled in this distance e-learning postgraduate programme did not actively participate in using a range of digital platforms and digital tools in their undergraduate programmes (Figure 5) with many unfamiliar with the full potential of PowerPoint and some students had never used this tool. Educators should, therefore, actively seek opportunities to embed digital skills and digital communication skills in both undergraduate [57] and postgraduate biomedical science degree programmes which are IBMS accredited and/or HCPC approved. Additionally, the use of digital tools in e-learning programmes, enhances engagement with the discipline specific content, provides motivation in relation to learning and assessment activities, and promotes communication between students and their peers as well as tutors [30]. Furthermore, from this study, the group-based activities fostered students’ confidence in taking on leadership roles and instilled an appreciation of the importance of inclusivity of other’s viewpoints and abilities, which aligns with the updated HCPC Standards of Proficiency in relation to equality, diversity and inclusion, namely, 5: “*recognise the impact of culture, equality and diversity on practice and practise in a non-discriminatory and inclusive manner*” [27].

### Study Limitations

The primary limitation to this study was the small number of students participating in each of the group assessment tasks. It must however be acknowledged that the number of enrolled students in each cohort did not exceed approximately 20 as this is a specialism module in an e-learning distance learning MSc programme. Additionally, uptake rates of the pre and post surveys particularly in relation to the second group assessment relating to the debate was low. This potentially was influenced by the fact that these students were active healthcare professionals delivering a microbiological diagnostic service during the peak of the COVID-19 pandemic and time was of a premium due to their healthcare, compulsory academic and personal commitments. It is widely reported the uptake rate of such online surveys is low, (30%–40%) [67], which is in the range of the second group activity survey responses (38% and 33% pre-and post-survey responses, respectively), with that of the first activity much higher (80% and 52% pre- and post-survey responses, respectively). This is unfortunately, a common fact of conducting surveys as has been highlighted recently by Bahir et al. [58]. In future questionnaires could be delivered using anonymous polls during live sessions by the digital tools such as Mentimeter [68] or incentives could be provided to increase uptake [58, 69]. Nevertheless, the embedding of these group assessments and their value to students in terms of developing transferable skills and building online communities is evident and the findings offer other educational providers an insight into novel approaches to developing group assessment in e-learning distance biomedical science degree programmes. Future work could focus on the co-creation of distance learning group activities, particularly debates, to address the challenges raised and promote further student engagement.

## Conclusion

In conclusion, online distance learning group activities are possible to implement if aligned with learning outcomes and clear instruction and support are provided. Although students and staff may find embedding such group assessments into the curriculum difficult, the benefit in terms of student experience is valuable. Students have an opportunity to build learning communities and have peer support, as well as develop specialist knowledge and key transferable skills, namely, digital competencies. Online debate formats improve students’ critical evaluation skills and promote a novel online approach to teaching and assessment which students find interesting and enjoyable.

## Summary Table

### What Is Known About The Subject?

• The availability of IBMS accredited biomedical science distance learning degree programmes is increasing.
• Distance learning students can often feel isolated due to the temporal separation from both their tutor and peers.
• The revised HCPC SOPs emphasise the need to be able to keep up-to-date with digital skills and new technologies.

### What This Paper Adds

• Group-based assessments improved students’ knowledge and development of digital skills.
• Completion of digital outputs from group-based activities were regarded as enjoyable experiences.
• Students developed transferable digital skills and confidence during online group activities, realising the importance of digital competencies in the healthcare sector.

## Summary Sentence

This work represents an advance in biomedical science because distance learning group assessments promoted online learning communities and the development of key transferable digital skills.

## Data Availability

The original contributions presented in the study are included in the article/Supplementary Material, further enquiries can be directed to the corresponding author.
