# Supplementary material for: Group Assessments to Help Build Online Learning Communities in Biomedical Science Distance Learning Programmes
Source: Br J Biomed Sci. 2023 Dec 15;80:11891. doi: 10.3389/bjbs.2023.11891 (PMC10754981; doi:10.3389/bjbs.2023.11891)
Supplement: Supplementary file 1 [file DataSheet2.PDF]

Supplementary Table 2: Self/peer marking marking template

Self/Peer Assessment of performance and participation to the team activity.

Please give a mark out of 5 for each aspect of the teamwork.
Maximum total mark would therefore be 25.

Enter each **Student Name (including yours)** in boxes below

Your Name:

Student Number:

Aspects of teamwork (Criteria)	**(Yourself)**	(Team member name)	(Team member name)	(Team member name)	(Team member name)
Was he/she regularly at group meetings, punctual & co-operative?					
Did he/she contribute ideas and suggestions for the project?					
How well did he/she carry out the tasks assigned by the group?					
Did he/she accept a fair share of the work?					
How would you rate his/her overall contribution to the project?					
**TOTAL FOR EACH STUDENT**					
