# Supplementary material for: Group Assessments to Help Build Online Learning Communities in Biomedical Science Distance Learning Programmes
Source: Br J Biomed Sci. 2023 Dec 15;80:11891. doi: 10.3389/bjbs.2023.11891 (PMC10754981; doi:10.3389/bjbs.2023.11891)
Supplement: Supplementary file 2 [file DataSheet4.PDF]

Supplementary Data File 4: SKILLS Post-Evaluation Questionnaire

	21st CENTURY SKILLS	1 Strongly Disagree	2 Disagree	3 Neither agree nor disagree	4 Agree	5 Strongly Agree
1.	I possess the skills and abilities that postgraduate level employers seek	1 ☐	2 ☐	3 ☐	4 ☐	5 ☐
2.	I have demonstrated **complex problem solving** during my assignment task	1 ☐	2 ☐	3 ☐	4 ☐	5 ☐
3.	I have developed and applied **critical thinking** during my assignment task	1 ☐	2 ☐	3 ☐	4 ☐	5 ☐
4.	I have developed **creativity** during my assignment task	1 ☐	2 ☐	3 ☐	4 ☐	5 ☐
5.	I have **coordinated with other people** during my assignment task	1 ☐	2 ☐	3 ☐	4 ☐	5 ☐
6.	I have used **digital media and digital communication tools** while completing my assignment task	1 ☐	2 ☐	3 ☐	4 ☐	5 ☐
	**DIGITAL SKILLS:** **CREATIVITY AND INNOVATION**	**1 Strongly Disagree**	**2 Disagree**	**3 Neither agree nor disagree**	**4 Agree**	**5 Strongly Agree**
7.	I possess the digital skills and abilities connected to digital creation that employers within the field of biomedical sciences seek from postpective employees	1 ☐	2 ☐	3 ☐	4 ☐	5 ☐
8.	I have demonstrated creativity and innovation through my assignment task	1 ☐	2 ☐	3 ☐	4 ☐	5 ☐
9.	I have applied existing knowledge to generate new ideas, products, or processes while completing my assignment task	1 ☐	2 ☐	3 ☐	4 ☐	5 ☐
10.	I have created original works using digital technologies about	1	2	3	4	5

	new ideas, projects and opportunities during my assignment task	☐	☐	☐	☐	☐
11.	I have captured, created and produced new digital materials such as digital stories and video during my assignment task	1 ☐	2 ☐	3 ☐	4 ☐	5 ☐
12.	I have shared and showcased digital artefacts with an awareness of audience and purpose during my assignment task	1 ☐	2 ☐	3 ☐	4 ☐	5 ☐

	**DIGITAL SKILLS:**<br><br>**COLLABORATION AND COMMUNICATION AND PARTICIPATION**	**1**<br>**Strongly Disagree**	**2**<br>**Disagree**	**3**<br>**Neither agree nor disagree**	**4**<br>**Agree**	**5**<br>**Strongly Agree**
13.	I have participated in a range of digital communication video and photo sharing, during my assignment task<br>**E-mail**<br>**On-line Discussion Forums**<br>**Slides sharing via Blackboard Ultra**<br>**On-line oral presentation**<br>**Video sharing**<br>**Photo sharing**<br>**File sharing**<br>**Shared Calendars**<br>**Webinars**	1<br>☐<br>☐<br>☐<br>☐<br>☐<br>☐<br>☐<br>☐<br>☐	2<br>☐<br>☐<br>☐<br>☐<br>☐<br>☐<br>☐<br>☐<br>☐	3<br>☐<br>☐<br>☐<br>☐<br>☐<br>☐<br>☐<br>☐<br>☐	4<br>☐<br>☐<br>☐<br>☐<br>☐<br>☐<br>☐<br>☐<br>☐	5<br>☐<br>☐<br>☐<br>☐<br>☐<br>☐<br>☐<br>☐<br>☐
14.	I have used a number of digital communication tools completing my assignment task, in a **respectful and inclusive way**	1 ☐	2 ☐	3 ☐	4 ☐	5 ☐

	EMPLOYABILITY	Not important	Minimal importance	Unsure	Slightly important	Very important
		1	2	3	4	5
15.	In your opinion, how important are developing your **work related skills** during postgraduate study to securing a postgraduate job in the biomedical science sector?	1	2	3	4	5
	**Communication**	☐	☐	☐	☐	☐
	**Team work**	☐	☐	☐	☐	☐
	**Leadership**	☐	☐	☐	☐	☐
	**Time management**	☐	☐	☐	☐	☐
	**Ability to work alone**	☐	☐	☐	☐	☐
	**Accuaracy**	☐	☐	☐	☐	☐
	**Confidentiality**	☐	☐	☐	☐	☐
	**Respecting individuals**	☐	☐	☐	☐	☐
	**Understanding Health & Safety**	☐	☐	☐	☐	☐
16	In your opinion, how important are developing your **work related skills** during post graduate study to job progression in the biomedical science sector?	1	2	3	4	5
	**Communication**	☐	☐	☐	☐	☐
	**Team work**	☐	☐	☐	☐	☐
	**Leadership**	☐	☐	☐	☐	☐
	**Time management**	☐	☐	☐	☐	☐
	**Ability to work alone**	☐	☐	☐	☐	☐
	**Accuaracy**	☐	☐	☐	☐	☐
	**Confidentiality**	☐	☐	☐	☐	☐
	**Respecting individuals**	☐	☐	☐	☐	☐
	**Understanding Health & Safety**	☐	☐	☐	☐	☐
17.	In your opinion, how important are developing your **digital skills** at university to securing a postgraduate job in the biomedical science sector?	1 ☐	2 ☐	3 ☐	4 ☐	5 ☐
18.	In your opinion, how important are digital skills to employers offering postgraduate jobs in the biomedical science sector?	1 ☐	2 ☐	3 ☐	4 ☐	5 ☐

19.	How likely are you to apply for job opportunities that state they require digital skills?	**Not Likely**	**Unsure**	**Likely**	**Very Likely**
		1 ☐	2 ☐	3 ☐	4 ☐
20.	How confident are you in talking about examples of using a range of digital media in job applications and interviews?	**Not confident**	**Slightly confident**	**Confident**	**Very confident**
		1 ☐	2 ☐	3 ☐	4 ☐

21. Had you previously used the Flip Grid digital tool?

Yes ☐ No ☐

What are your opinions of the Flip Grid digital tool?

Thank you for taking the time to complete this questionnaire.
