# Supplementary material for: Group Assessments to Help Build Online Learning Communities in Biomedical Science Distance Learning Programmes
Source: Br J Biomed Sci. 2023 Dec 15;80:11891. doi: 10.3389/bjbs.2023.11891 (PMC10754981; doi:10.3389/bjbs.2023.11891)
Supplement: Supplementary file 3 [file DataSheet3.PDF]

Supplementary Data File 3: SKILLS PRE-Evaluation Questionnaire

	21st CENTURY SKILLS	**1** **Strongly Disagree**	**2** **Disagree**	**3** **Neither agree nor disagree**	**4** **Agree**	**5** **Strongly Agree**
1.	I possess the skills and abilities that graduate level employers seek	1 ☐	2 ☐	3 ☐	4 ☐	5 ☐
2.	I have demonstrated **complex problem solving** during my **previous undergraduate** course	1 ☐	2 ☐	3 ☐	4 ☐	5 ☐
3.	I have demonstrated **complex problem solving** since commencing my **current post-graduate** course	1 ☐	2 ☐	3 ☐	4 ☐	5 ☐
4.	I have applied **critical thinking** during my **previous undergraduate**	1 ☐	2 ☐	3 ☐	4 ☐	5 ☐
5.	I have applied **critical thinking** during my **current post-graduate course**	1 ☐	2 ☐	3 ☐	4 ☐	5 ☐
6.	I have developed **creativity** during my **previous undergraduate**	1 ☐	2 ☐	3 ☐	4 ☐	5 ☐
7.	I have developed **creativity** during my **current post-graduate course**	1 ☐	2 ☐	3 ☐	4 ☐	5 ☐
8.	I have **coordinated with other people** during my **previous undergraduate** course	1 ☐	2 ☐	3 ☐	4 ☐	5 ☐
9.	I have **coordinated with other people** during my **current post-graduate course**	1 ☐	2 ☐	3 ☐	4 ☐	5 ☐
10.	I have used **digital media and digital communication tools** during my **previous undergraduate** course	1 ☐	2 ☐	3 ☐	4 ☐	5 ☐
11.	I have used **digital media and digital communication tools** during my **current post-graduate course**	1 ☐	2 ☐	3 ☐	4 ☐	5 ☐

	**DIGITAL SKILLS:**<br><br>**CREATIVITY AND INNOVATION**	**1**<br>**Strongly Disagree**	**2**<br>**Disagree**	**3**<br>**Neither agree nor disagree**	**4**<br>**Agree**	**5**<br>**Strongly Agree**
12.	I possess the digital skills and abilities connected to digital creation that graduate level employers seek	1<br>☐	2<br>☐	3<br>☐	4<br>☐	5<br>☐
13.	I have demonstrated creativity and innovation through my current course at university	1<br>☐	2<br>☐	3<br>☐	4<br>☐	5<br>☐
14.	I have applied existing knowledge to generate new ideas, products, or processes while on my current course	1<br>☐	2<br>☐	3<br>☐	4<br>☐	5<br>☐
15.	I have captured, created and produced new digital materials such as digital stories and video on my current course	1<br>☐	2<br>☐	3<br>☐	4<br>☐	5<br>☐
16.	I have shared and showcased digital artefacts with an awareness of audience and purpose on my current course	1<br>☐	2<br>☐	3<br>☐	4<br>☐	5<br>☐

	**DIGITAL SKILLS:**<br><br>**COLLABORATION AND COMMUNICATION AND PARTICIPATION**	**1**<br>**Strongly Disagree**	**2**<br>**Disagree**	**3**<br>**Neither agree nor disagree**	**4**<br>**Agree**	**5**<br>**Strongly Agree**
17.	I have participated in a range of digital communication video and photo sharing, during my **previous undergraduate course**<br><br>**E-mail**<br>**On-line Discussion Forums**<br>**Slides sharing via Blackboard Ultra**<br>**On-line oral presentation**<br>**Video sharing**<br>**Photo sharing**<br>**File sharing**<br>**Shared Calendars**<br>**Webinars**	1<br>☐<br>☐<br>☐<br>☐<br>☐<br>☐<br>☐<br>☐<br>☐	2<br>☐<br>☐<br>☐<br>☐<br>☐<br>☐<br>☐<br>☐<br>☐	3<br>☐<br>☐<br>☐<br>☐<br>☐<br>☐<br>☐<br>☐<br>☐	4<br>☐<br>☐<br>☐<br>☐<br>☐<br>☐<br>☐<br>☐<br>☐	5<br>☐<br>☐<br>☐<br>☐<br>☐<br>☐<br>☐<br>☐<br>☐

18.	I have participated in a range of digital communication video and photo sharing, during my **current postgraduate course**					
		1	2	3	4	5
	**E-mail**	☐	☐	☐	☐	☐
	**On-line Discussion Forums**	☐	☐	☐	☐	☐
	**Slides sharing via Blackboard Ultra**	☐	☐	☐	☐	☐
	**On-line oral presentation**	☐	☐	☐	☐	☐
	**Video sharing**	☐	☐	☐	☐	☐
	**Photo sharing**	☐	☐	☐	☐	☐
	**File sharing**	☐	☐	☐	☐	☐
	**Shared Calenders**	☐	☐	☐	☐	☐
	**Webinars**	☐	☐	☐	☐	☐
	**EMPLOYABILITY**	**Not important**	**Minimal importance**	**Unsure**	**Slightly important**	**Very important**
		1	2	3	4	5
19.	In your opinion, how important are developing your **work related skills** during postgraduate study to securing a postgraduate job in the biomedical science sector?	1	2	3	4	5
	**Communication**	☐	☐	☐	☐	☐
	**Team work**	☐	☐	☐	☐	☐
	**Leadership**	☐	☐	☐	☐	☐
	**Time management**	☐	☐	☐	☐	☐
	**Ability to work alone**	☐	☐	☐	☐	☐
	**Accuaracy**	☐	☐	☐	☐	☐
	**Confidentiality**	☐	☐	☐	☐	☐
	**Respecting individuals**	☐	☐	☐	☐	☐
	**Understanding Health & Safety**	☐	☐	☐	☐	☐

20.	In your opinion, how important are developing your **work related skills** during post graduate study to job progression in the biomedical science sector?	1	2	3	4	5
	**Communication**	☐	☐	☐	☐	☐
	**Team work**	☐	☐	☐	☐	☐
	**Leadership**	☐	☐	☐	☐	☐
	**Time management**	☐	☐	☐	☐	☐
	**Ability to work alone**	☐	☐	☐	☐	☐
	**Accuaracy**	☐	☐	☐	☐	☐
	**Confidentiality**	☐	☐	☐	☐	☐
	**Respecting individuals**	☐	☐	☐	☐	☐
	**Understanding Health & Safety**	☐	☐	☐	☐	☐
21.	In your opinion, how important are developing your **digital skills** at university to securing a postgraduate job in the biomedical science sector?	1<br>☐	2<br>☐	3<br>☐	4<br>☐	5<br>☐
22.	In your opinion, how important are digital skills to employers offering postgraduate jobs in the biomedical science sector?	1<br>☐	2<br>☐	3<br>☐	4<br>☐	5<br>☐

123.	How likely are you to apply for job opportunities that state they require digital skills?	**Not Likely**	**Unsure**	**Likely**	**Very Likely**
		1<br>☐	2<br>☐	3<br>☐	4<br>☐
24.	How confident are you in talking about examples of using a range of digital media in job applications and interviews?	**Not confident**	**Slightly confident**	**Confident**	**Very confident**
		1<br>☐	2<br>☐	3<br>☐	4<br>☐

Thank you for taking the time to complete this evaluation questionnaire.
