# Supplementary material for: Group Assessments to Help Build Online Learning Communities in Biomedical Science Distance Learning Programmes
Source: Br J Biomed Sci. 2023 Dec 15;80:11891. doi: 10.3389/bjbs.2023.11891 (PMC10754981; doi:10.3389/bjbs.2023.11891)
Supplement: Supplementary file 4 [file DataSheet1.pdf]

Supplementary Table 1

Learning outcomes of "Advances in Medical Microbiology" 30-credit module

Successful students will be able to:

1 Critically evaluate the changing diagnostic role of the clinical microbiology laboratory particularly in light of developments in automation and molecular methods

2 Evaluate reasons for the changing epidemiology of infectious diseases and areas of global concern, including antimicrobial resistance, bioterrorism and emerging/re- emerging pathogens, through both independent examination of the scientific literature and group-based learning.

3 Retrieve, interpret and critically appraise research-based findings relating to key concepts and hypotheses of pathogenic mechanisms, and appreciate how this knowledge can be utilised within the field of translational clinical microbiology to ensure that appropriate diagnostic and therapeutic approaches are used to effectively treat and control infection.

4 Develop and appreciate the importance of transferable soft and digital skills for use in the workplace. Develop and appreciate the importance of good teamworking skills to address the real-world challenges of creating a piece of work worthy of publication in a peer reviewed scientific journal or oral / poster presentation at a national / international conference.
