# Supplementary material for: Group Assessments to Help Build Online Learning Communities in Biomedical Science Distance Learning Programmes
Source: Br J Biomed Sci. 2023 Dec 15;80:11891. doi: 10.3389/bjbs.2023.11891 (PMC10754981; doi:10.3389/bjbs.2023.11891)
Supplement: Supplementary file 5 [file DataSheet5.PDF]

Supplementary Data File 5: Skills relating to securing a job and job progression

A: Importance of developing work-related skills during post graduate study to secure a job in the biomedical science sector

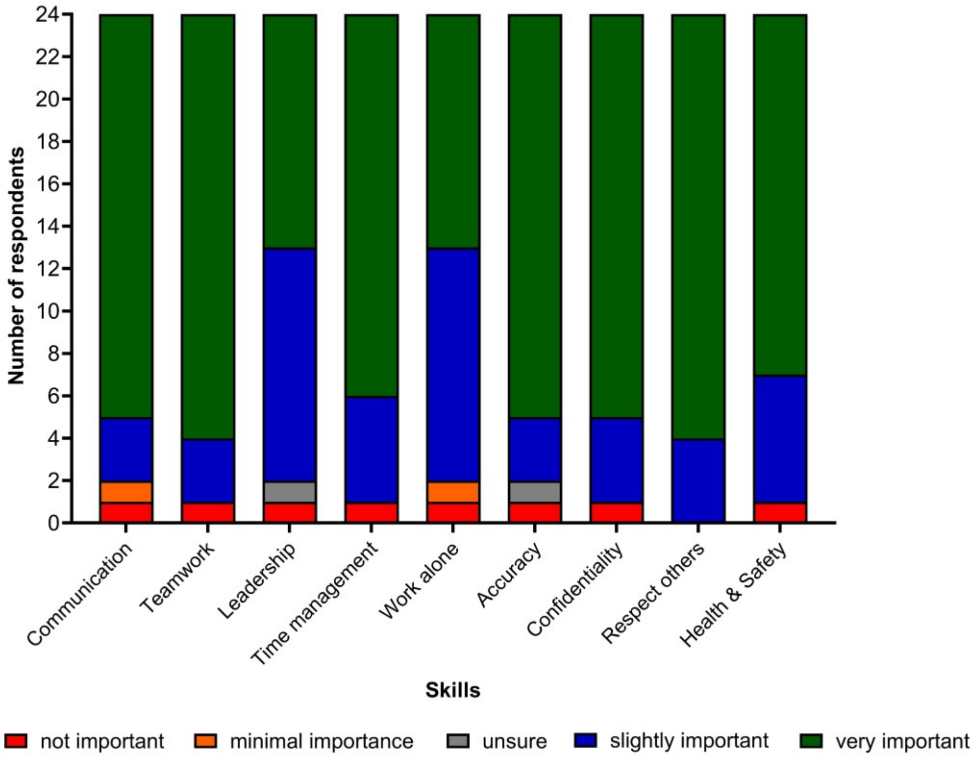

B: Importance of developing work-related skills during post graduate study in relation to job progression in the biomedical science sector

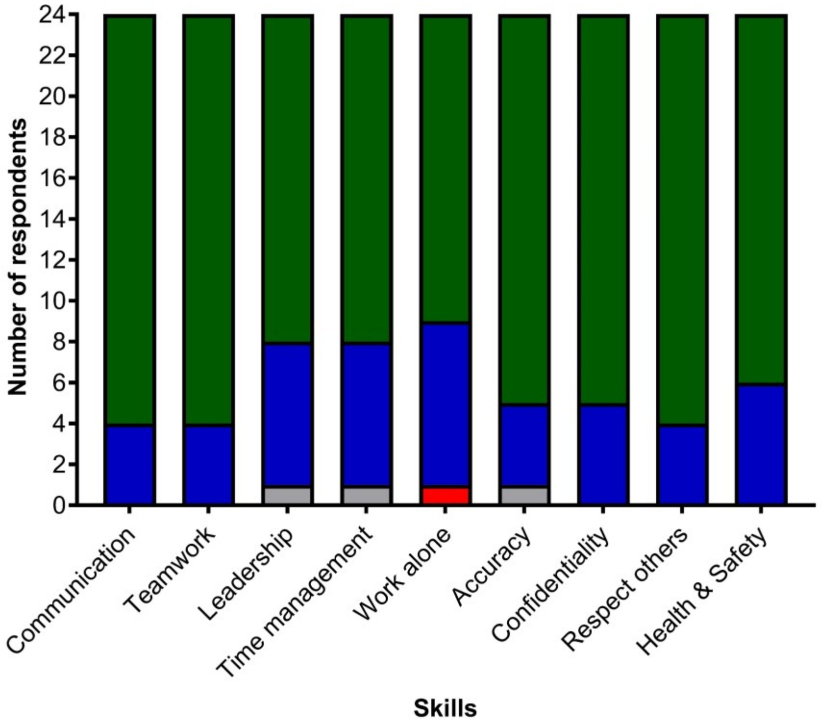

N.B. No statistical difference was noted either between skills nor between securing a job and job progression
